# A Novel Approach for Automated Analysis of Cell Attachment and Spreading Based on Backscattered Electron Imaging by Scanning Electron Microscopy

**DOI:** 10.3390/ma2031402

**Published:** 2009-09-24

**Authors:** Alisa Katsen-Globa, Leonora Peter, Susan Zöllner, Thomas Dörge, Martin Daffertshofer, Hartwig Preckel, Daniel Schmitt, Heiko Zimmermann

**Affiliations:** 1 Fraunhofer IBMT, Ensheimer Str.48, 66386 St.Ingbert, Germany; E-Mails: alisa.katsen@ibmt.fraunhofer.de (A.K.); leonora.peter@ibmt.fraunhofer.de (L.P.); susan.zoellner@ibmt.fraunhofer.de (S.Z.); thomas.doerge@ibmt.fraunhofer.de (T.D.); daniel.schmitt@ibmt.fraunhofer.de (D.S.); 2 Perkin Elmer Cellular Technologies GmbH, Schnackenburgallee 114, 22525 Hamburg, Germany; E-Mails: martin.daffertshofer@perkinelmer.com (M.D.); hartwig.preckel@perkinelmer.com (H.P.)

**Keywords:** cell adhesion, cell spreading, cytocompatibility of materials, cell/material interactions, scanning electron microscopy, backscattered electron imaging, image analysis

## Abstract

The development of new materials for biological application requires *in vitro* testing of cell/surface interactions. Cell adhesion and spreading are difficult to quantify as most materials are non-transparent and transmission microscopy cannot be used. Contrast in reflection microscopy is rather poor. We propose an alternative method for the automated screening of cell attachment and spreading using backscattered electron imaging of scanning electron microscopy. The enhanced cell contrast permits study of cell/material interactions by little differences between cells and material.

## 1. Introduction 

The development of biotechnology requires the screening and testing of new materials and the search for new assays of cyto-, and biocompatibility [1]. Prominent examples are surfaces of implants, *in vitro* culture devices and new innovative applications like surface based freezing [2]. This keeps a cell in its natural environment without loss of proliferation or differentiation. Two different issues need to be addressed. Firstly, the thermophysical properties of materials must be suitable for low temperature applications. Secondly, the materials must be compatible with the cells being preserved. In this paper we have tested as candidates for cryopreserving adherent cells, two widely used materials: polyethylene and aggregated carbon nanotubes. 

Cell adhesion and spreading have been studied on surfaces with different topographies for the last 40 years [3,4,5,6,7,8]. Topographical properties of materials can induce various cell reactions such as cell adhesion, spreading, motility, proliferation and death [9,10,11,12,13,14,15,16,17,18]. Slight differences in topography including nano-topography cause changes in cell spreading [13,14,19,20,21,22,23] and produce a wide variety of cellular responses including up- and down regulation of different genes [14,23]. Most of these effects can be detected only after days of cell cultivation. Whereas cell attachment and spreading can be observed after a few some hours [24]. 

The quantification of cell spreading is a key aspect of cell/material research [1,25,26,27,28]. Most methods are based on light microscopy, e.g., IRM, TIRFM, SPRM, etc. [25] and have used fluorescence immunolabeling of cytoskeleton and focal adhesion sites [15,19,20,21]. Sensitivity of fluorescence labeling is limited by bleaching [1], and it is difficult to distinguish cell contours to quantify cell spreading area. High-resolution methods, such as scanning electron microscopy (SEM), can improve visualization and thus quantification of the adhesion/spreading area of single cells [4,26,27,28,29,30,31,32]. Such methods are time consuming and necessitate the investigation of high numbers of individual cells at high magnification for valid statistical conclusions. In this work we present a new method for automated, quick, high throughput scanning electron microscopic screening of a large number of spread cells in area of interest by analysis a backscattered electron (BSE) images. Such analysis was performed with commercial available image processing system. In this paper we concentrate on the workflow and potential of the method rather than on precise cell/material interactions.

## 2. Results and Discussion 

### 2.1.Tested materials 

**Figure 1:**
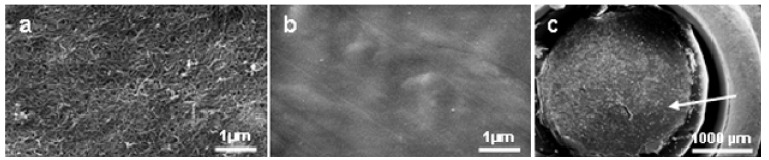
Rough - and smooth surfaces for cell cultivation. (a) carbon (C) nanotubes surface. (b) high density polyethylene (PE)-surface. (c) C-nanotubes (arrow) mounted on the bottom of the wells of the PE-microsubstrate for cell cryopreservation.

We have tested two materials with different topographical and chemical properties: carbon (C) sheets [see Figure 1(a)], produced as “buckypaper” from C-nanotubes and high density polyethylene (PE)–microsubstrate [see Figure 1(b)]. PE-microsubstrates were produced at Fraunhofer IBMT for cell cryopreservation [2]. C-substrates for cell cultivation were punched out and mounted on the bottom of PE-microsubstrates for following cell cultivation [Figure 1(c)].

The materials have different surface topography: rough, non-ordered C-nanosurface [Figure 1(a)] and smooth, almost planar PE-surface [Figure 1(b)]. Roughness can affect cell adhesion and spreading [24,31,32,33]. However, its measurement with a special device [33] was not a focus of this paper. 

To evaluate the surface hydrophobicity/hydrophilicity of the tested materials a measurement of contact angles was performed [34]. Both materials were originally hydrophobic, as confirmed by water their contact angle measurements (about 78°), and therefore not suited for cell attachment. Argon and oxygen plasma treatments were used to make the surfaces hydrophilic. After plasma treatment, the contact angle for PE was between 8° and 13° within the first hour and slowly increased to 19° over the next 24 hours. Similar results were obtained for C-nanotubes (data not shown). 

Plasma treatment, a widely used process in biomedical engineering [35], increases hydrophilicity. Both oxygen and argon plasmas remove contamination (argon by ablation, oxygen chemically) and may improve cell attachment [35]. PE may become more hydrophilic due to slight adsorption of molecules on the polymer surface allowing production of extra-cellular matrix (ECM) after cell adhesion and spreading [14]. We have not observed significant differences between inert argon plasma and reactive oxygen plasma in surface topography of both materials and cell behavior (see results below). The relative importance of plasma cleaning and chemical modification needs to be confirmed with other sensitive methods, such as surface x-ray photoelectron spectroscopy [34,36]. 

### 2.2. Cell response to chosen materials 

**Figure 2:**
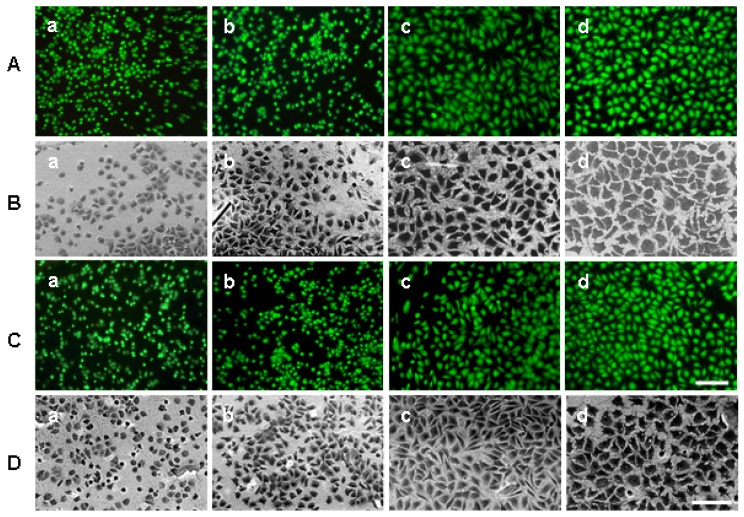
Representative inverted fluorescence (rows A and C) and BSE-microscopic images (rows B and D) of L929 cells after cultivation on the C-surface. The cells were cultured for 2 (a), 4 (b), 8 (c) and 24 (d) hours on C-surface treated with argon-plasma (A and B) or oxygen-plasma (C and D). Scale bars: 100 μm.

**Figure 3:**
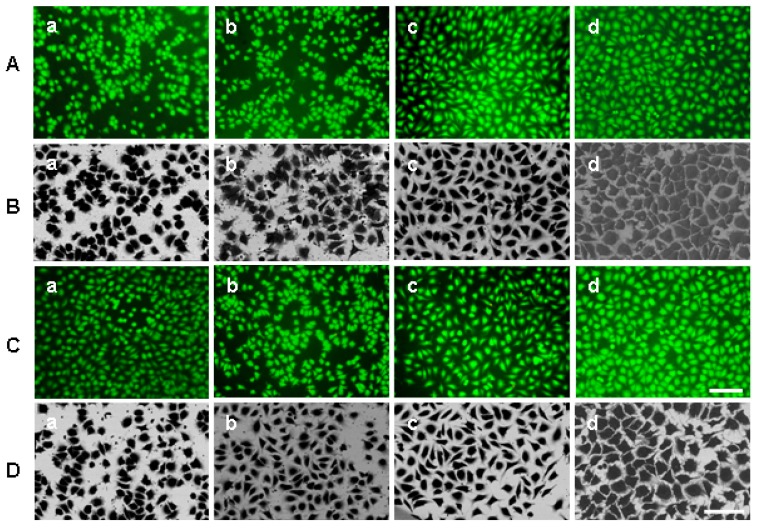
Representative inverted fluorescence (rows A and C) and BSE-microscopic images (rows B and D) of L929 cells after cultivation on the PE-surface. The cells were cultured for 2 (a), 4 (b), 8 (c) and 24 (d) hours on PE-surface treated with argon-plasma (A and B) or oxygen-plasma (C and D). Scale bars: 100 μm.

Combined fluorescence microscopy and SEM of the same preparations demonstrates that L929 cells can attach to both substrates and remain vital after cultivation up to 24 hours (Figure 2, Figure 3). 

Representative images of live green fluoresced cells are shown in rows A and C of Figure 2 (C-surface), and Figure 3 (PE-surface). The viability/membrane integrity test demonstrated that both substrates were cytocompatible for L929 cells after plasma treatment. More than 90% of the attached cells were vital (data not shown). Not all cells were in focus by fluorescence imaging [for example, Figure 2A(a); Figure 3A(a)] due to, perhaps, a slight curvature of both substrates and some cell detachment at the beginning of cultivation. It makes the exact calculation of cell number difficult. 

**Figure 4:**
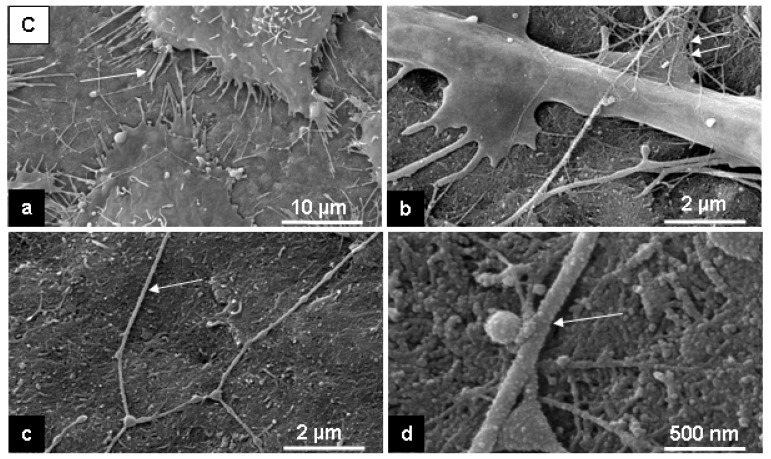
Representative SE-microscopic images of cell behavior on the C-nanostructured surfaces (nanotubes) after 24 hours of cultivation. Note: cell filopodia by cell-substrate contacts (a, arrow), cell traces (c and d, arrow) and a fine filaments network on the substrate and over the cell surface (b, double arrows).

**Figure 5:**
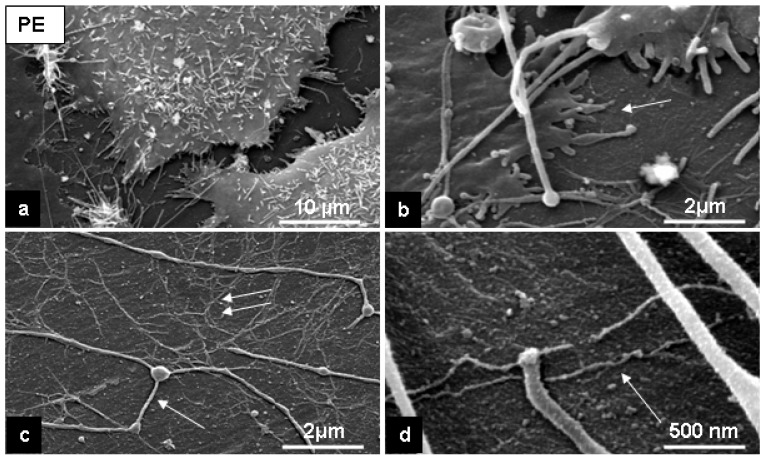
Representative SE-microscopic images of cell behavior on the PE-surface after 24 hours of cultivation. Note: a flattening of cells (a), cell filopodia by cell-substrate contacts (b, arrow), cell prints of motility (c, arrow) and a fine filaments network on the substrate (c, double arrows, d, arrow).

SEM in the secondary electron (SE)-mode revealed that cells had adhered to the both substrates and built cell-to-cell and cell-to-substrate contacts. This is clearly seen after 24 hours of cultivation (Figure 4, a, b and d; Figure 5, a, b). Cell morphology observed with SEM showed that L929 fibroblasts were well spread on the flat PE-surface and on the rough surfaces of C-nanotubes (Figure 4, a, and Figure 5, a). 

We have observed various cell numbers in fluorescence and SEM-images of the same preparations (Figure 2 and Figure 3, compare rows A and B, C and D). Some cells were probably detached during re-washing from fluorescent stains before fixation and can be interpreted as preparation artefacts. 

After 24 hours cultivation on the C-, and PE-surfaces we have observed with SEM thin fibers, about 150-200 nm in diameter [Figure 4 (c and d), arrow; Figure 5(c), arrow]. We also found a fine filamentous network directly attached to the C-nanotubes surface and PE-surface [Figure 4(b); Figure 5(c), double arrows]. On the PE-surface, we observed a thin layer of small beads [<100 nm; Figure 5(c and d)] that was not present on the original surface [Figure 1(b)]. This probably consists of weakly adsorbed serum proteins that enable cell adhesion and spreading [33]. 

The cells can move on the substrates, and the features of cell motility, i.e., cell traces, studied and classified by Zimmermann *et al*. [37], were also observed on the investigated surfaces [see Figure 4(c and d); Figure 5(c), arrow]. Fine filaments with a diameter of about 100 nm were seen on the both substrates and sometimes over the cell surfaces, but predominantly on the PE-surface [see Figure 4(b), Figure 5(c), double arrows]. These filaments may correspond to the structures expressed by fibroblasts (extracellular matrix proteins following cell adhesion) [14,22]. This needs to be confirmed with immunolabelling .

### 2.3. Quantification of cell attachment & spreading 

Representative inverted BSE-images of the spread cells studied with a viability/membrane integrity test are shown in rows B and D of Figure 2 and Figure 3. BSE-images confirmed that the number and spread of cells differed on the chosen substrates. The brightness and contrast of these images changes with the topography of substrates and the number of adherent cells. 

**Figure 6:**
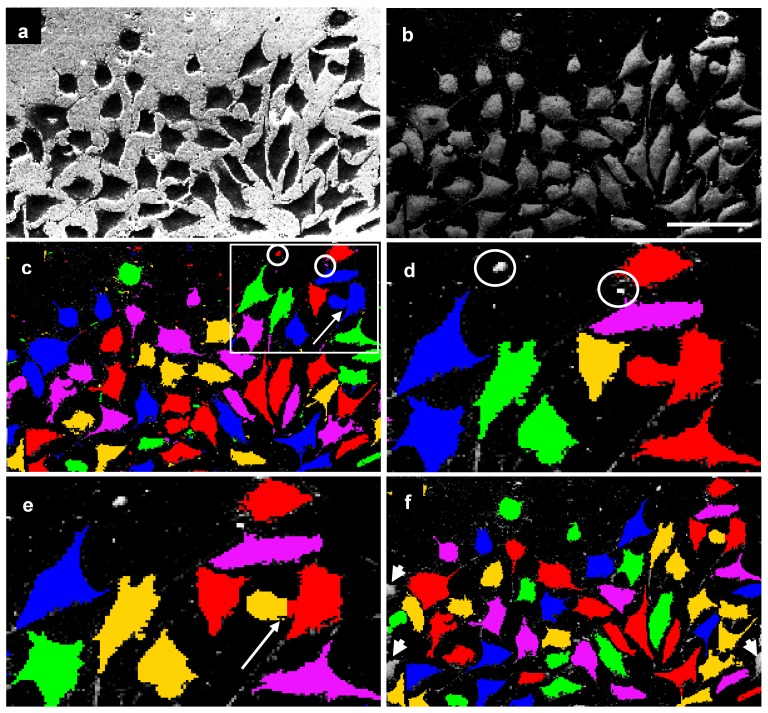
Image processing steps of BSE-images; (a) original image; (b) subtraction of background information after image inversion; (c) threshold segmentation: rectangle marked part of image shown in d and e; (d)-trouble shooting (objects marked in c and d with circles); (e) splitting of cells by distance (cells marked in c and e with arrow); (f) final image after removing of fragmentary border cells (arrow heads). Scale bar: 100 μm.

**Figure 7:**
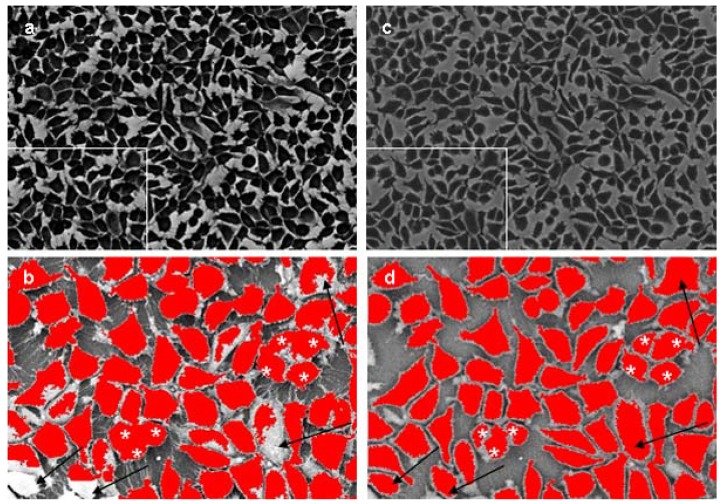
Automated image processing of SE- and BSE- images of the same preparation; (a) inverted original SE –image; (b) processed rectangle marked in (a) part of SE- image; (c) original BSE- image; (d) processed rectangle marked in (c) part of BSE-image. Note the recognition of whole cell area (d, arrows) and cell splitting (d, asterisks) in the BSE image in contrast to only a partial identification of cell area (b, arrows) and no cell splitting (b, asterisks) in SE.

The algorithm of cell spreading quantification is presented in the Experimental section. The image processing reliably found the whole cell area (excluding some fine cell protrusions) and marked it for measurement [Figure 6(f) - last image]. With SE-images as input, the program did not recognize and mark the whole cell area correctly [Figure 7(b), arrows] and could not split tangent cells after threshold segmentation [Figure 7(b), asterisks]. 

**Figure 8:**
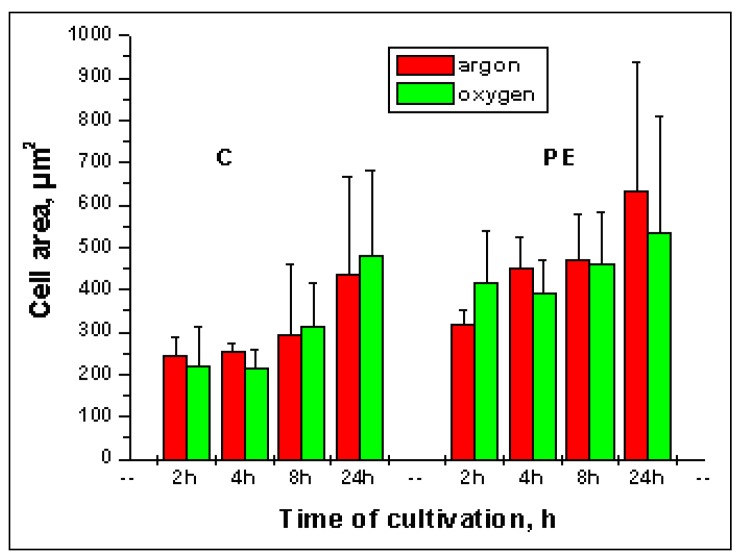
Comparison of cell spreading by cultivation on the C-, and PE-surfaces treated with argon and oxygen plasma. There were no statistically significant differences between the two plasma treatments (p < 0.05). The cells are less well spread on the C-surface than on the PE-surface.

Some results of quantitative analysis of cell spreading are shown in Figure 8. Data represent the mean of three experiments each four replicates ± standard deviation. The difference between experiments analyzed using Student`s test was not random (p < 0.05). Quantification of cell area (Figure 8) has indicated that: (a) cell area increases with a time in both substrates; (b) spreading area of L929 fibroblasts on the C-nanotubes surface was less than PE-surface in each time of cultivation; (c) there were no significant differences between argon and oxygen plasma treatments for any surface. 

**Figure 9:**
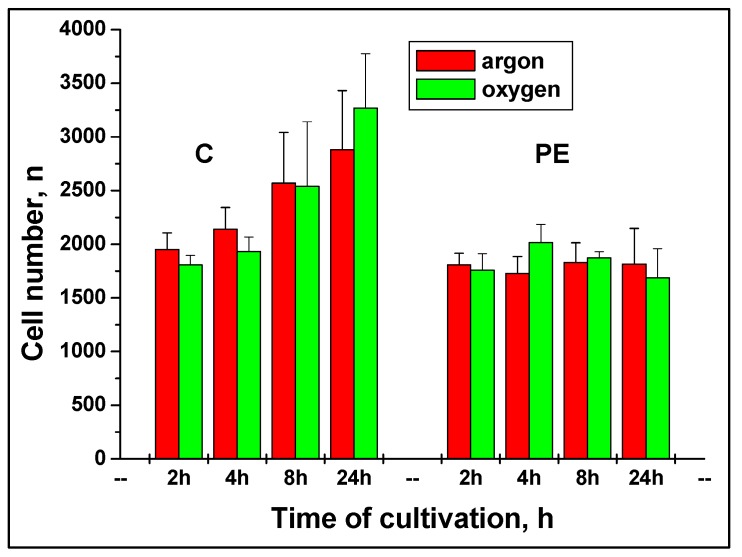
Mean cell number of calculated attached cells after cultivation on the C-, and PE-surfaces treated with argon and oxygen plasma. Cell number was calculated by BSE- imaging. There were no statistically significant differences between two plasma treatments (p < 0.05).

We have also calculated a mean cell number from BSE-images (Figure 9). More cells were attached to the C-nanotubes surface than to PE-surface, and the number of cells has increased with time of cultivation. The number of cells attached to the PE-surface was nearly constant for both plasma treatments for the whole time course of experiments.

The present data indicated some variation in cell behavior on the different materials. The number of attached cells differed despite there being the same number of seeded cells. The high number of cells initially attached to C-nanotubes might be explained by increased adhesion strength due to the surface roughness in comparison to the almost smooth PE-surface [33,38]. This hypothesis could be investigated by measuring the correlation of attachment with average surface roughness amplitude [24,33]. 

Spreading area increased with time on both substrates and was higher on PE. It is well known that cell adhesion is mediated by formation of focal contacts [1,3]. Possibly, L929 cells attach to carbon with a small number of focal adhesion sites while the PE-surface promotes cell adhesion and spreading. Increased protein adsorption at the material surface and increased expression of extracellular matrix proteins may be responsible, as suggested by investigation of direct cell adhesion to polymeric surfaces [33].

We have tested cell behaviour on the nanostructured (C) and planar (PE) surfaces. Cell spreading on the nanotopographical surface is reported to be reduced in comparison with planar surface [39]. In our experiments the differences in spreading area were significant but not high. One possible reason is that C-surface was almost planar („buckypaper”) but still nanostructured. 

After 24 h of cultivation on the both substrates standard deviations by measuring of cell area were high. Most likely, some cells has already divided that reduced cell surface area [40] and affected on the measured values.

### 2.4. Advantages of developed method

#### 2.4.1. Contrast optimization of BSE-images 

The proposed method is based on SEM in the BSE-mode. To get optimal contrast for BSE-images, we have treated the cells with heavy metals and tannic acid [41,42]. A similar strategy has been also used for contrast optimization of BSE-images of resin-embedded cells [29,30]. In our scheme, the tannic acid used between osmium tetroxide and uranyl acetate, serves as a link between two metals with a high atomic number - osmium and uranium – and, therefore, enhances the image contrast of cells by depositing heavy metals. This facilitates studies with weak contrast between cell and substrate (for example cells spreading on metal implants). This treatment also improved the conductance of the samples, enabling SE-imaging of cells on PE substrates coated with only a thin layer of carbon without charging. 

#### 2.4.2. Automated BSE-imaging analysis 

The method is also based on automated analysis of spread cells in an area of interest using the Acapella Image Analysis Program. The same images were also verified with the NI Vision Development Module and gave similar results (data not shown). Unlike other software for cell spreading analysis [8,14,15,19,20,23,27], this program can automatically recognize and process cells, correct backgrounds and remove all non-cellular objects. Most of the above-cited approaches have used light (fluorescence) microscopic images for quantitative analysis. However, fluorescence microscopic images cannot yield the total cell areas due to a limited resolution and relatively quick fluorochrome bleaching [1]. Using BSE-imaging SEM improves investigation and image processing of spread cells [7,26,30]. In these studies, cells were manually marked for the calculation of areas. The advantages of our method are the automated marking of objects, the enhanced contrast of cells achieved with heavy metal and tannic acid impregnation together with a background subtraction [Figure 6(b)]. It is especially beneficial for variable brightness of C-substrates (Figure 2, B, C). Some fine cell protrusions have not been recognized with the current script. This small part of cell area did not affect the results of high throughput screening of cell spreading on the various materials.

## 3. Experimental Section

### 3.1. Substrate preparation 

Cells were cultured on the following substrates: high density polyethylene (PE)–microsubstrate [see Figure 1(b)] for cell cryopreservation produced at Fraunhofer IBMT [2] and carbon (C) nanotubes (Nanoledge, Montpellier, Cedex, France), produced as “buckypaper” [Figure 1(a)]. Punched out C-nanotubes were mounted [Figure 1(c)] on the bottom of the PE-substrate wells (3 mm in diameter). PE-substrate was cleaned with 70% ethanol, C-nanotubes were autoclaved. After ethanol-cleaning, the substrates were treated with argon or oxygen plasma (0.8 mbar, 75 W, 120 s) in Diener electronic equipment (Germany), then full-blown 60s with N_2_ gas and finally UV-irradiated for 15 minutes. Water contact angle was measured to indicate the hydrophilicity of the substrates. The experiments were started within 1 hour of UV-irradiation.

### 3.2. Cell culture

L929 mouse fibroblasts were cultured in Dulbecco's modified growth medium with 10% foetal bovine serum and 100 µg/mL gentamycin and incubated at 37 °C under 5% carbon dioxide/ 95% air. All substances were obtained from PAN Biotech GmbH (Germany). The cells were seeded on both substrates at a concentration of 2 × 10^5^ cells/mL. The PE-substrate was regarded as a control. 

### 3.3. Cell viability/Membrane integrity test 

At different times during culture (2, 4, 8 and 24 hours), the cells were stained using a cell viability/membrane integrity test. This test uses the enzyme substrate fluorescein diacetate (FDA) and DNA-dye ethidium bromide (EB) [43]. Both fluorescent dyes were obtained from Sigma (USA). The viability fluorescence assay was examined with an SMZ 1500 Zoom Stereomicroscope with Epifluorescence attachment (Nikon, Japan) and photographed with a Coolpix 5000 digital camera (Nikon, Japan). Fluorescent images were processed with the Acapella Image Analysis Program (PerkinElmer, Cellular Technologies Germany GmbH, Hamburg). 

### 3.4. Scanning electron microscopy (SEM)

After viability testing, the substrates with cells were washed, fixed and treated for SEM with osmium tetroxide, tannic acid and uranyl acetate as previously described [41,42]. Then the cells were dehydrated in increasing series of ethanol and critical point dried in an automated Polaron Range critical point dryer CPD 7501 (Quorum Technologies Ltd.). The substrates with cells were coated with carbon in SCD-030 (Balzers, Lichtenstein) or gold-palladium in a Polaron high resolution sputter coater SC 7640 (Quorum Technologies Ltd.) and examined in a field emission scanning electron microscope FESEM XL30 (Phillips, USA) or variable pressure scanning electron microscope LEO 435 VP (Carl Zeiss NTS GmbH, Oberkochen, Germany). An accelerating voltage 5 and 10 kV was used for secondary electron (SE) imaging. Cell spreading was studied using backscattered electron (BSE) modes with 15 kV accelerating voltage, 243 pA probe emission current and 12 mm working distance for all images.

### 3.5. Quantification of cell spreading 

For quantitative analysis of cell spreading, the electron microscopic images were taken in BSE-mode or by the mixing of BSE and SE in ratio 90:10 at low magnification (200×) without tilting the object table. To better distinguish cell contours, we have inverted the images. 

Each substrate was investigated three times after 2, 4, 8, 24 hours of cell cultivation. In each experiment at least four images from different regions of substrate and a total number of at least 300 cells were analyzed. Image processing was implemented in a scripting language of Acapella Image Analysis Program (PerkinElmer, Cellular Technologies Germany GmbH, Hamburg). The different steps of processing are shown in Figure 6. Processing of the original image [Figure 6(a)] involves the following algorithm:

1. Creation of an inverse image.
2. Generation of background image information and its subtraction from the original image. In processing a non-uniform background, a smooth brightness gradient can be subtracted out. To generate an image with only this gradient, a mean filter with a large mask across a whole image was optimal [Figure 6(b)].
3. Application of threshold segmentation. After this, discrete pixels were clustered to cell objects (marked by different colors in Figure 6). The cells were added to object lists for further processing steps. The magnitude of threshold segmentation was automatically adapted to the particular images.
4. Deletion of non-cell objects. Small blemishes were automatically removed using a size filter [Figure 6(c and d), objects marked with circles].
5. Splitting of tangent cells by distance. In some cases, threshold segmentation cannot separate several tangent cells [Figure 6(c and e), arrow]. An algorithm based on evaluation of the distance of each point in the cell interior to the nearest cell border was used to split those cells.
6. Removal of fragmentary border cells. Border cells with area located predominantly outside the image (above half area) were checked and removed [Figure 6(f)].
7. Calculation of several figures of merit like cell count, total area of cells, etc.

Using this program we have also compared SE- and BSE-image processing of the same microscopic field (Figure 7). Cell spreading was estimated, with the same software, as the mean area of one cell (Figure 8) and calculated as the measured area of the cells in the images divided by the number of cells. Taking into account the pixel resolution and the scale of images the cell area in µm² was determined. A mean value of whole cell number in BSE-images was calculated and considered as cell number in Figure 9. 

### 3.6. Statistical analysis

Student’s t-test for dependent values was used to determinate a significance of mean area and a mean cell number at each time point of cell cultivation. For comparison of two plasma treatment groups, the non-parametric Wilcoxon signed rank test was applied. The statistical difference between C-nanosurface and PE-surface was estimated with a Welch test. Results of p < 0.05 were considered significant.

## 4. Conclusions 

In this paper we present a new method for investigation and quantification of cell attachment and spreading on the non-transparent materials. The method based on the high-resolution contrast backscattered electron images made with scanning electron microscopy and automatically analyzed. The method can distinguish well differences between cells and surfaces, as well as differences between various materials. This method can be used in material sciences for testing of cyto-, bio-, and cryocompatibility of materials. 
